# Collage and Assemblage in the Microbial World

**DOI:** 10.3201/eid1410.000000

**Published:** 2008-10

**Authors:** Polyxeni Potter

**Affiliations:** Centers for Disease Control and Prevention, Atlanta, Georgia, USA

**Keywords:** Art science connection, emerging infectious diseases, art and medicine, Eric Mack, Atlanta art, collage and assemblage, APMR-41553, microbial adaptability, about the cover

**Figure:**
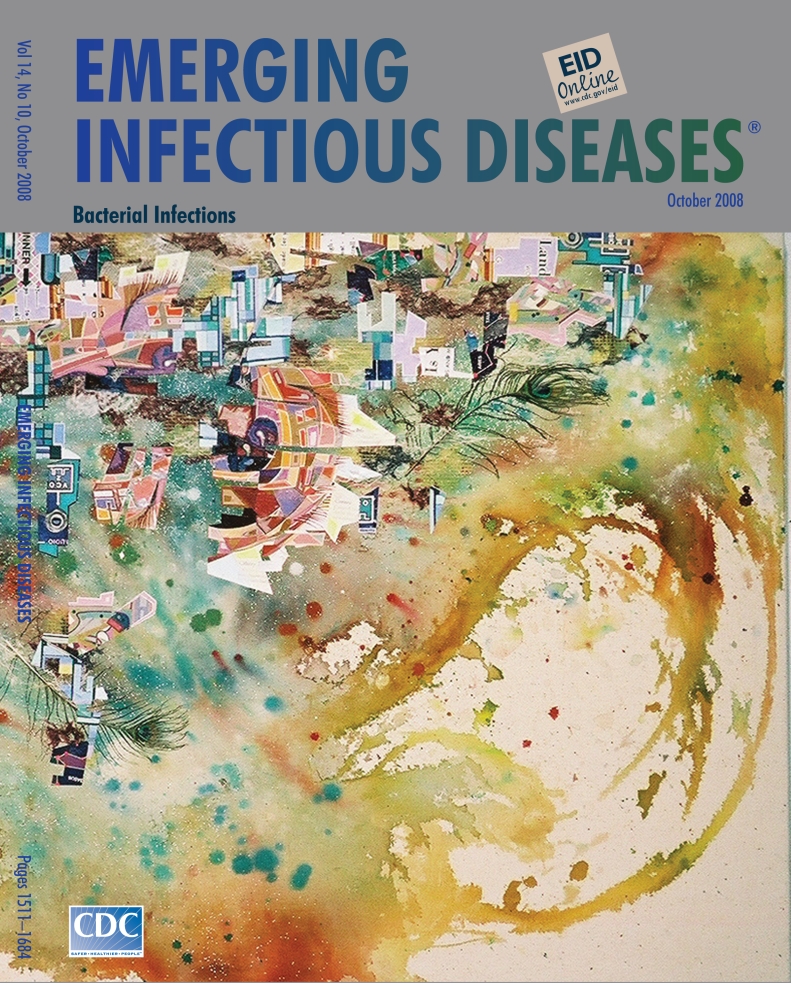
**Eric Mack (b. 1976) APMR-41553 (2007)** Mixed media on canvas (182.8 cm × 91.4 cm) Used with permission of the artist. Photo by Fay Gold Gallery, Atlanta, Georgia, USA

“There are two problems in painting” said American artist Frank Stella (b. 1936) when he was still a brash young man. “One is to find out what painting is, and the other is to find out how to make a painting” (*1*). Stella’s interest in the definition of painting may have reflected late 19th- early 20th-century modernist concerns. More contemporary concerns focus primarily on how to make a painting, how to use materials, methods, concepts, or traditions to create a work that may not even be called a painting. *How* a work is determines *what* it is (*2*). Atlanta artist Eric Mack, a man of his times, is part of the contemporary scene and its pressures to create a painting.

At one time, art was organized by schools, later by movements. Today, these designations are difficult to apply. Painting cannot be categorized exclusively as a certain palette or style, and no consistent look or theme can be used to quantify its sum. The old labels (abstract, realist, symbolist, surrealist, expressionist, narrative) may still describe some works. But others defy all convention. They have no images, no drawing, no color, no canvas, or are not made by the artist’s hand.

“I’m all about shape, pattern, and repetition of form,” is how Mack describes his own work (*3*). Among his techniques, collage and assemblage are suited for discovering new forms and forming new ways to connect objects retrieved from the world. His canvases contain synthetic netting, natural fibers, leaves, Japanese text, board game graphics, corporate logos, small levers and sockets, plugs, dials and switches. These bits and pieces of objects, punctuated by a painted eyeball here, a peacock feather there, are laboriously integrated into a fluid backdrop of color applied by brush or aerosol. The effect refers to recent traditions, among them abstract expressionism, pop, and graffiti (*4*).

“She got me those clippers and told me that once I learned to cut hair,” Mack says of his mother, a cosmetologist, “I would never have to worry about going hungry.” His career as barber flourished throughout his years at the Atlanta College of Art, when he was co-owner of “Barber’s Edge” on Buford Highway, the city’s bustling multiethnic corridor where he tended all styles and customers: “Flattops, fades, skin fades, shadow fades, babies to old people, black, white, Chinese, Mexican, Puerto Ricans, Cubans, Africans, anyone who has hair.” But his artistic career started much earlier, “I remember different breakdancing moves like the Windmill that you could do. I’d draw people doing the dance moves and color them up and sell them for, like, a dollar or two dollars in elementary school” (*5*).

Mack’s American childhood, begun with his birth in suburban Charleston, South Carolina, was otherwise unremarkable. Yearly trips to New York to visit family exposed him to music, which would become a major inspiration. As an art student, he met and was influenced by another Atlanta artist, Charles Nelson, who encouraged him to move from illustration to painting. “I was too intimidated by the brush. Once I took a chance and tried it out, I thought ‘This brush is like the clippers.’ It came like I had been doing it for a long time” (*5*). He joined a group of local artists, among them Kevin Cole and Kojo Griffin, exhibited his work often, and became a fixture of the Atlanta art scene.

APMR-41553 on this month’s cover of Emerging Infectious Diseases embodies the sprawl, clutter, and flux of today’s human communities. A complex composite of unlikely pieces, it portrays new forms constructed out of old and attached as never before.

An organic arrangement of viable human, mechanical, and environmental parts, the work has rich metaphorical potential. The solid microcosm anchored against undulating fluid, now blotted with clumps of color, now disappearing against bare canvas, encompasses all, including human and microbial interaction.

“Our relationship to infectious pathogens,” wrote the late Joshua Lederberg, “is part of an evolutionary drama. Here we are. Here are the bugs” (*6*). These ubiquitous bugs have, among other advantages, the ability to change in ways that make them dangerous. For example *E*. *coli* O157:H7 started out as a pathogen capable of causing mild diarrhea. Newly acquired genes transformed it into a virulent microbe that also destroys kidneys and erythrocytes (*7*). Diversification in some strains of group A streptococci, common bacteria that normally do not cause disease in humans, is causing the reemergence of a severe form of invasive disease (*8*).

Collage and assemblage, which work so well in Mack’s art, mirror microbial activity that can cause havoc in the global community. Like the artist’s bits of objects, microbial ultrastructures can reassort, recombine, and reassemble into brand new entities. They adapt to new ecologic niches or species, produce new toxins, and bypass or suppress immune defenses to infect humans and animals. Their plasticity frustrates vaccine development, and they become resistant to even the most potent drugs.

For all its apparent fragmentation, Mack’s vision is universal and positive. The reassembled objects form a new working composite. But the microbial equivalent is still a puzzle seeking solution in science’s mixed media: new technologic bits and pieces (vaccines, drugs, diagnostic tools) arranged against crowding and social, political, and economic stratification.

## References

[1] Stella F. Text of a lecture at the Pratt Institute. In: The writings of Frank Stella. Cologne: Verlag der Buchhandlung Walther König; 2001.

[2] Schwabsky B. Vitamin P: new perspectives in painting. Boston: Phaidon Press; 2004.

[3] Fox C. Interview with Eric Mack. Atlanta Journal-Constitution. Arts section; 2003 June 1.

[4] Thompson J. How to read a modern painting: lessons from the modern masters. New York: Harry N. Abrams, Inc.; 2006.

[5] Feaster F. A study in abstraction. Creative Loafing. 2002;31:28.

[6] Lederberg J. Emerging infections: an evolutionary perspective. Emerg Infect Dis. 1998;4:366–71. 9716947 10.3201/eid0403.980306 PMC2640283

[7] Grant J, Wendelboe AM, Wendel A, Jepson B, Torres P, Smelser C, Spinach-associated *Escherichia coli* O157:H7 outbreak, Utah and New Mexico, 2006. Emerg Infect Dis. 2008;14:1633–6. 10.3201/eid1410.071341 18826833 PMC2609868

[8] Aziz RK, Kotb M. Rise and persistence of global M1T1 clone of *Streptococcus pyogenes.* Emerg Infect Dis. 2008;14:1511–7. 10.3201/eid1410.071660 18826812 PMC2609857

